# Iodoform Paste

**Published:** 1888-11

**Authors:** R. D. Pedley


### Iodoform Paste.

In filling the roots of teeth whose nerves have undergone decomposition, the following is a most excellent paste:

| | |
|---|---|
| Ol. eucalypti - - - - | 2 parts |
| Ol. caryoph - - - - - - | 3 parts |
| Creosote - - - - - - | 10 parts |

Into this gum mastic should be dissolved to saturation. After filtering through cotton-wool, thoroughly incorporate the solution with iodoform in a wedgewood mortar until it becomes almost a solid mass. The oil keeps the preparation moist; creosote to a certain extent disguises the smell of the iodoform; the gum holds it well together, and one is enabled to introduce about twice as much as when used dry. Introduce with wisps of cotton and over it place a disc of wood or metal. The preparation undergoes no change in or out of the mouth.

R. D. Pedley, L.D.S.

*D. Record*, Sept.

